# Sanyin Formula Enhances the Therapeutic Efficacy of Paclitaxel in Triple-Negative Breast Cancer Metastases through the JAK/STAT3 Pathway in Mice

**DOI:** 10.3390/ph16010009

**Published:** 2022-12-22

**Authors:** Chunyu Wu, Chenping Sun, Xianghui Han, Yiyi Ye, Yuenong Qin, Sheng Liu

**Affiliations:** 1 Department of Breast Surgery (Integrated Traditional and Western Medicine), Longhua Hospital, Shanghai University of Traditional Chinese Medicine, 725 South Wanping Road, Shanghai 200032, China; 2 Longhua Hospital, Institute of Chinese Traditional Surgery, Shanghai University of Traditional Chinese Medicine, 725 South Wanping Road, Shanghai 200032, China

**Keywords:** Sanyin formula (SYF), triple-negative breast cancer (TNBC), signal transducer and activator of transcription 3 (STAT3) pathway, cancer cell metastasis, network pharmacology

## Abstract

Sanyin formula (SYF) is used as a complementary treatment for triple-negative breast cancer (TNBC). The purpose of this study was to identify the potential functional components and clarify the underlying molecular mechanisms of SYF in TNBC. High-performance liquid chromatography–tandem mass spectrometry (HPLC-MS/MS) was used to identify the main components of SYF extracts. Network pharmacology and bioinformatic analyses were carried out to identify potential candidate targets of SYF in TNBC. Cell proliferation was determined with a Celigo imaging cytometer. Wound-healing and Transwell assays were adopted to evaluate cell migration. A Transwell cell-invasion assay was performed with Matrigel-coated membranes. In vivo bioluminescence imaging (BLI) and pathological analyses illustrated the effect of SYF on cancer cell metastasis in tumour-bearing mice. The inhibitory mechanism of SYF was investigated via quantitative PCR (qPCR) and Western blotting. We found that 3,4-dihydroxyphenyllactic acid, kaempferol, p-coumaric acid, and vanillic acid may be the active components of SYF. Molecular docking confirmed that kaempferol, p-coumaric acid, vanillic acid, and 3,4-dihydroxyphenyllactic acid bound stably to proteins such as AKR1C3, MMPs, and STAT3. SYF extract suppressed TNBC cell proliferation, migration, invasion, and metastasis by inhibiting JAK/STAT3 signalling and then regulating downstream genes, such as MMP-2/MMP-9. SYF regulates the expression of genes involved in cell proliferation, migration, and invasion by regulating the JAK/STAT3 signalling pathway and finally inhibits tumour cell metastasis in TNBC. The present study clarifies the mechanism by which SYF inhibits TNBC metastasis and lays an experimental foundation for the continued clinical development of SYF targeting the JAK/STAT3 pathway.

## 1. Introduction

In 2020, the number of breast cancer patients exceeded the number of lung cancer patients for the first time, and breast cancer became the most frequently diagnosed cancer [1]. As one of the molecular subtypes of breast cancer, although triple-negative breast cancer (TNBC) only accounts for 15–20% of all breast cancer cases, its prognosis is the worst, especially in the advanced or metastatic patients [2,3]. This is mainly due to the lack of oestrogen receptor (ER), progesterone receptor (PR), and human epidermal growth factor receptor 2 (HER2) in these TNBC patients, which directly leads to unresponsiveness to conventional antihormonal therapy and anti-HER2-targeted treatments [4]. Additionally, TNBC has a higher recurrence rate than other types of breast cancer [5]. Conventional chemotherapy and surgery are currently the recommended treatments for TNBC [6,7]. Unfortunately, the current treatment options for TNBC have limited efficacy, and the promising targeted therapies and immunotherapies are still not clinically approved [8]. Therefore, TNBC remains the most intractable aggressive subtype of breast cancer [9].

In China, traditional Chinese medicine is widely used in the complementary treatment of cancers and is recognized by doctors and patients [10]. A meta-analysis indicated that traditional Chinese medicine combined with Western medicine has more obvious advantages than Western medicine alone in the treatment of TNBC patients [11]. Sanyin formula (SYF), a commonly used supplementary traditional Chinese medicinal formula, was found to effectively reduce recurrence and metastasis in TNBC patients [12,13]. Our previous prospective cohort study indicated that SYF significantly increased the 2-year disease-free survival (DFS) rate and reduced the disease-related recurrence and metastasis rate by 11.0% in TNBC patients [12]. In our recent randomized controlled trial, the 5-year DFS time was longer in those who received SYF compared with placebo (94.2% vs. 85.5%, *p* = 0.034) [13]. These results indicated that SYF could significantly reduce tumour cell metastasis in TNBC. However, SYF is composed of nine traditional Chinese medicines and contains dozens of compounds; thus, identifying the main active components of SYF is difficult. In addition, the molecular mechanism underlying the inhibitory effect of SYF in TNBC remains unknown. Therefore, the present study aimed to initially identify the main components and candidate targets of SYF by integrating high-performance liquid chromatography–tandem mass spectrometry (HPLC-MS/MS) analysis results with search results from the Therapeutic Target Database (TTD), the Traditional Chinese Medicine Systems Pharmacology Database and Analysis Platform (TCMSP), and the DisGeNET database and then to finally reveal the molecular mechanism by which SYF inhibits TNBC cell metastasis via bioinformatic enrichment analyses and experimental verification.

## 2. Results

### 2.1. Main Compounds in SYF Extract

**Figure 1:**
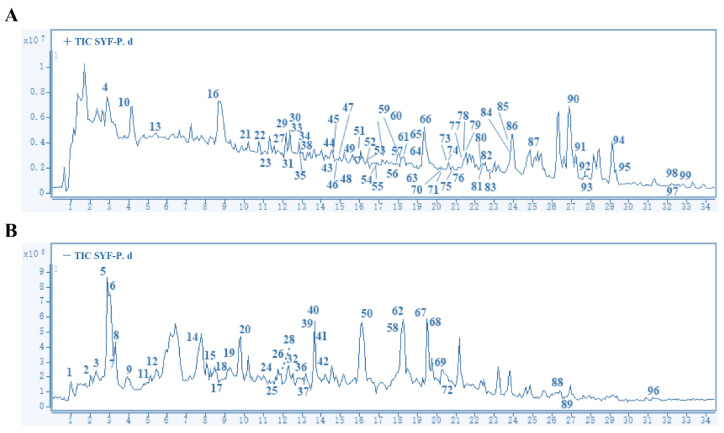
Total ion chromatogram of SYF as determined by HPLC-MS/MS. Positive ion mode (**A**) and negative ion mode (**B**). The X axis represents retention time (min), and the Y axis represents ionic strength.

A total of 99 peaks corresponding to the main compounds were identified by HPLC-MS/MS (Figure 1). After removing the repeated components, a total of 89 compounds or their isomers were identified (Supplementary Table S1). Codonopsine, 5-hydroxymethylfuraldehyde, magnoflorine, furanodiene, protocatechualdehyde, mannopyranoside, curcumenone, emodin, apigenin, icariin, kaempferol, p-coumaric acid, vanillic acid, etc., were the main compounds, and all had high relative contents. These compounds were extracted from *Atractylodes macrocephala* Koidz., *Scutellariae barbatae* D. Don, *Codonopsis pilosula* Nannf., *Curcuma phaeocaulis* Valeton, *Salviae chinensis* Herba, and *Epimedium brevicornu* Maxim. (Supplementary Table S1).

### 2.2. STAT3 Signalling Was Identified as the Potential Pathway

**Figure 2:**
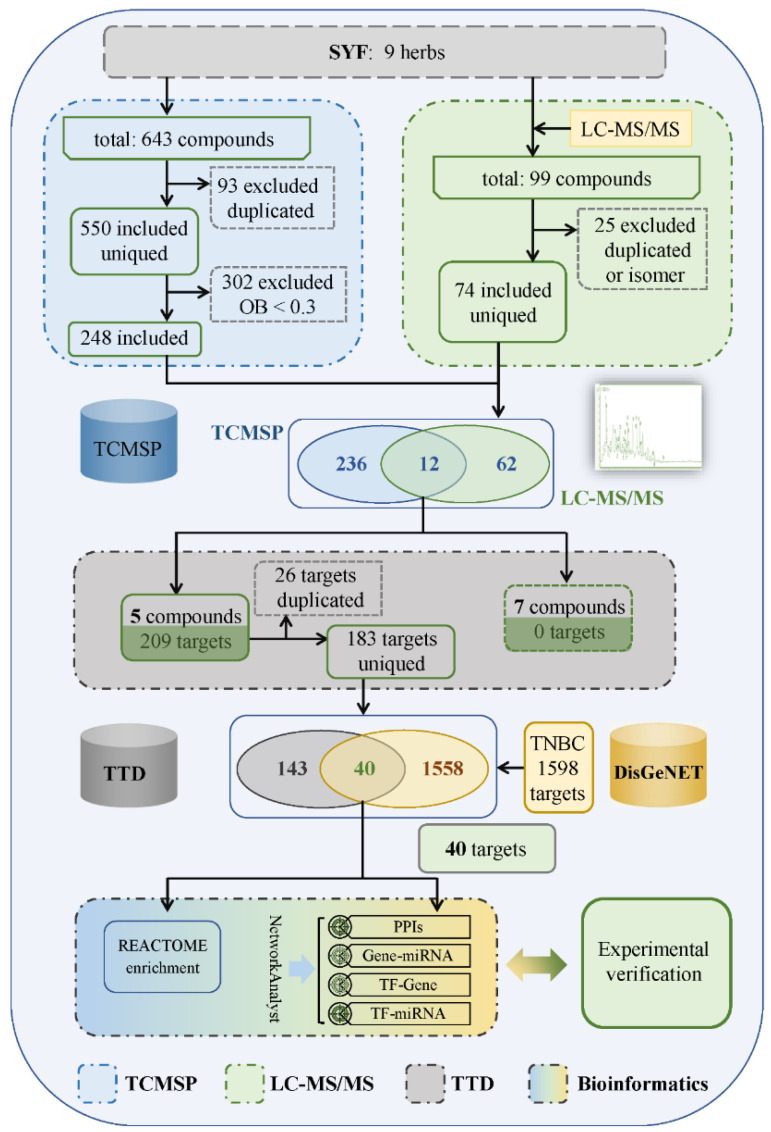
Flowchart of the network pharmacology and bioinformatic analysis approach.

In addition, the TCMSP database was used to identify the chemical constituents of SYF (Figure 2). A total of 550 components were identified after removing the repeated components. Subsequently, 248 components were identified by screening with the criterion of oral bioavailability (OB) > 0.3. A total of 12 chemical components were identified from the Venn diagram constructed with the results of HPLC–MS/MS analysis. A total of 183 potential targets were identified by searching these components in the TDD database. Finally, 40 candidate targets were identified from the Venn diagram constructed with the 1598 TNBC targets in the DisGeNET database (Supplementary Table S2).

**Figure 3:**
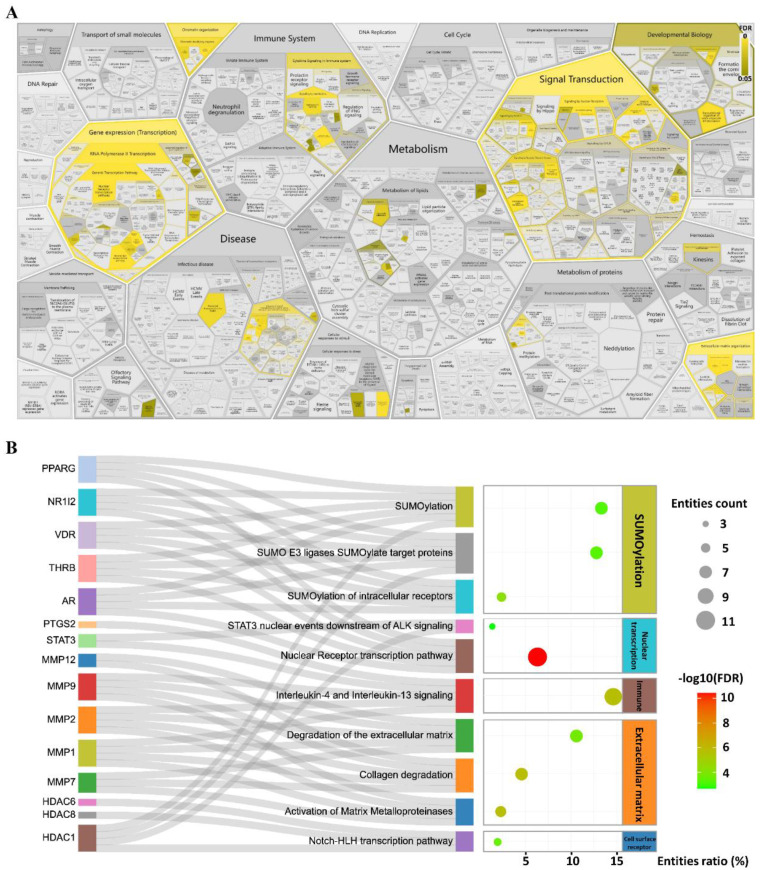
REACTOME enrichment results. (**A**) Overall map of enrichment results. (**B**) Top 10 signalling pathways based on increasing FDR and the genes involved in these pathways. The 40 candidate genes were used for REACTOME enrichment analysis at https://reactome.org (version 79, accessed on 13 March 2022).

Numerous REACTOME signalling pathways were enriched (Figure 3A). These differential pathways were distributed mainly in processes such as signal transduction, gene expression (transduction), extracellular matrix organization, immune system, and chromatin organization. Among them, 65 were significantly differentially enriched. SUMOylation, extracellular matrix, cell surface receptor, and nuclear-transcription-related pathways were the top-ranked signalling pathways (Figure 3B), such as collagen degradation, SUMOylation, STAT3 nuclear events downstream of ALK signalling, etc. *MMP-1*, *MMP-2*, *MMP-7*, *MMP-9*, *MMP-12*, *STAT3*, and *PYGS2* et al. genes were involved in the regulation of these pathways (Figure 3B).

**Figure 4:**
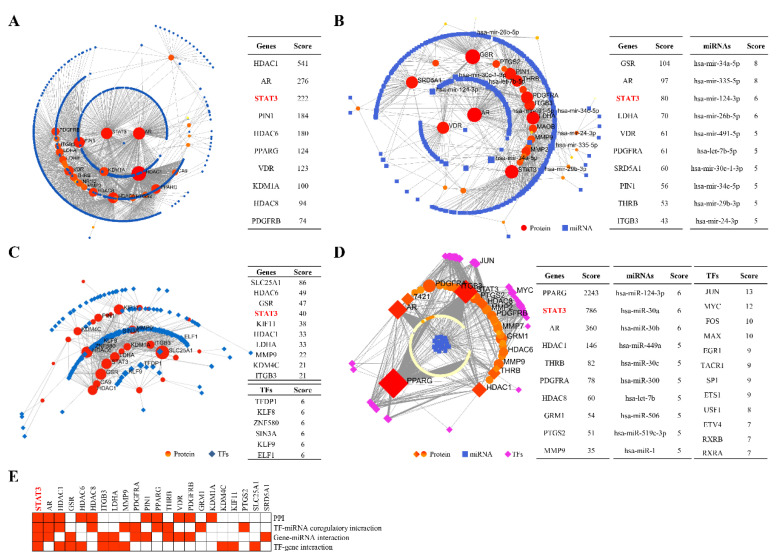
Interactions among genes, regulators, and signalling pathways. The 40 candidate genes were used for interaction analysis. Interactions among genes, TFs, miRNAs, and signalling pathways were analysed using NetworkAnalyst. (**A**) PPIs. The dots were proteins. (**B**) Gene-miRNA interactions. (**C**) TF-gene interactions. (**D**) TF-miRNA coregulatory interactions. (**E**) Ranking of potential key genes.

We then analysed the interactions among genes, regulators, and signalling pathways using NetworkAnalyst. Numerous interactions were identified in these networks (Figure 4). HDAC1, AR, and STAT3 interacted with other proteins in the PPI network (Figure 4A). The miRNAs hsa-miR-34a-5p, hsa-miR-335-5p, hsa-miR-124-3p, etc., regulated the expression of *GSR*, *AR*, and *STAT3*, etc. (Figure 4B). The TFs TFDP1, KLF8, ZNF580, etc., regulated the expression of *SLC25A1*, *HDAC6*, *GSR*, *STAT3*, etc. (Figure 4C). The miRNAs has-mir-124-3p, has-mir-30a, etc., and the TFs JUN, MYC, etc., coregulated the expression of *PPARG*, *STAT3*, *AR*, etc. (Figure 4D). Among these interactions, *STAT3* was the top-ranked gene and was identified as the potential key target gene using inductive analysis of these top-ranked genes (Figure 4E).

### 2.3. SYF Extract Reduced Cancer Cell Growth, Migration, and Invasion In Vitro

**Figure 5:**
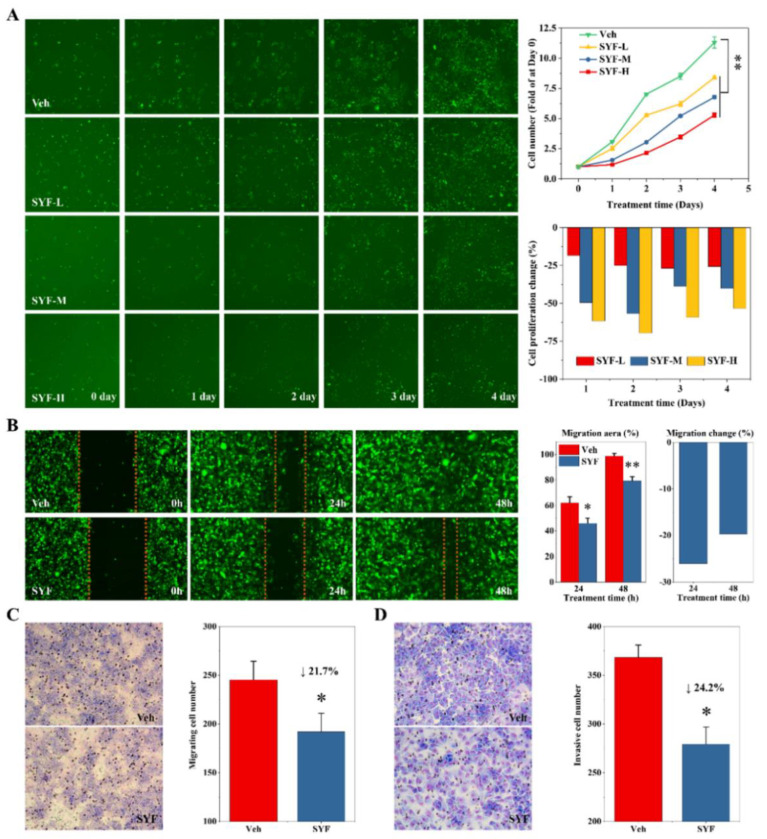
SYF inhibited breast cancer cell proliferation, migration, and invasion. (**A**) SYF inhibited breast cancer cell proliferation. After MDA-MB-231 cells were treated with different concentrations of SYF (L, 50 μg/mL; M, 100 μg/mL; H, 200 μg/mL) or vehicle (Veh), cells were photographed for 5 consecutive days, and cell growth curves were drawn to show cell proliferation after 5 days of consecutive recording using a Celigo imaging cytometer. (**B**) SYF (100 μg/mL) inhibited breast cancer cell migration, as determined using a wound-healing assay. Scratches were made with a wounding replicator, and the cells were photographed under a light microscope at the indicated times. The cell migration speed and inhibition rate were calculated as described in the Materials and Methods section. (**C**) SYF inhibited breast cancer cell migration. Cells were seeded at a density of 0.5 × 10^4^ cells/well in FBS-free medium in the upper chamber of a 24-well Transwell plate. The medium in the lower chamber contained 20% FBS. After the cells were incubated with or without SYF extract (100 μg/mL) for 48 h, the number of migrated cells was recorded with a Celigo imaging cytometer following staining with Giemsa (50× magnification) after removal of nonmigrated cells. (**D**) SYF inhibited breast cancer cell invasion using Transwell chambers containing Matrigel-matrix-coated membranes. Cells were seeded at a density of 1 × 10^4^ cells/well in FBS-free medium in the upper chambers of a 24-well Transwell plate containing Matrigel-precoated membranes. The medium in the lower chamber contained 20% FBS. After the cells were incubated with or without SYF extract (100 μg/mL) for 48 h, the number of migrated cells was recorded with a Celigo imaging cytometer following staining with Giemsa (50× magnification) after removal of nonmigrated cells. All experiments in this figure were performed in biological triplicate. * *p* < 0.05, ** *p* < 0.01 vs. Veh group.

We then analysed the effects of SYF on breast cancer cells. The results of the cell-growth assay using the Celigo system indicated that SYF significantly inhibited cancer cell proliferation in a dose-dependent manner (Figure 5A). The cell proliferation inhibition rate was approximately 20% in the low-dose extract group and approximately 40% in the middle- and high-dose groups (Figure 5A). The wound-healing assay results suggested that SYF significantly reduced the migration of MDA-MB-231 cells (Figure 5B). The cell migration inhibition rate was approximately 20% after SYF treatment for 48 h (Figure 5B). Similarly, consistent results were obtained in the Transwell assays (Figure 5C). In addition, the results of the Transwell assay with the Matrigel matrix indicated that SYF significantly inhibited breast cancer cell invasion (Figure 5D). It should be noted that at the later stage of the experiment, the cells in the control group almost reached their peak and did not continue to increase due to the tolerance limit of the experimental system. Therefore, the objective inhibition rate of SYF extracts on TNBC cells is higher than the current data.

### 2.4. SYF Extract Reduced Cancer Cell Metastasis In Vivo

**Figure 6:**
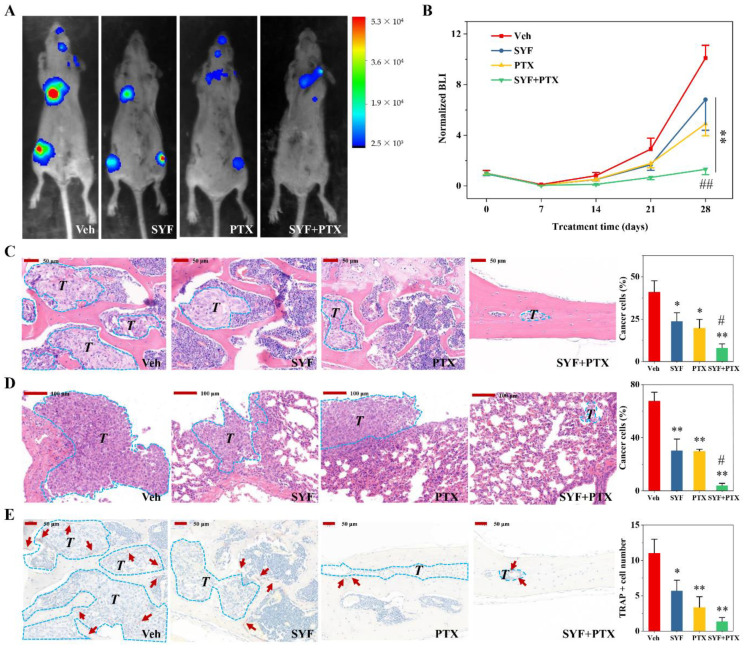
SYF reduced breast cancer cell metastasis in vivo. (**A**) SYF significantly reduced tumour cell metastasis in tumour-bearing mice. Tumour metastasis was achieved in mice by injection of breast cancer cells (1 × 10^6^ cells/mouse) into the left ventricle. SYF or vehicle was then administered daily, and paclitaxel was administered via intraperitoneal injection twice a week for 4 weeks (N = 6). In vivo BLI with a luciferase reporter was performed at the indicated times to assess the extent of tumour metastasis. (**B**) Normalized BLI signals. (**C**) Representative photographs of HE staining and quantitative analysis of tumour proportions in bone. (**D**) Representative photographs of HE staining and quantitative analysis of tumour proportions in lung metastases. (**E**) Representative photographs of TRAP staining and quantitative analysis of TRAP^+^ cells in bone. TRAP^+^ cells are marked with red arrows. Tumour metastases are marked with rose red dotted lines and the letter *T*. * *p* < 0.05, ** *p* < 0.01 vs. Veh group. ^#^ *p* < 0.05, ^##^ *p* < 0.01 vs. PTX group.

To evaluate the effects of SYF extract on cancer cell metastasis in mice, tumour-bearing mice were treated with SYF extract or vehicle for 4 weeks, and cancer metastasis was monitored using bioluminescence imaging (BLI). SYF significantly reduced the degree of tumour metastasis in mice compared with that in the vehicle group (Figure 6A,B). Interestingly, SYF significantly increased the efficacy of paclitaxel (PTX) (Figure 6A,B). The pathological HE staining results also confirmed that SYF significantly reduced tumour proliferation in bone metastases and lung metastases in tumour-bearing mice (Figure 6C,D). Additionally, SYF significantly reduced TRAP-positive cells in bone metastases (Figure 6E). SYF plus PTX combinatorial therapy also enhanced PTX’s inhibition of breast cancer metastasis and bone destruction (Figure 6C–E).

### 2.5. Candidate Compounds Bound Tightly to Candidate Target Proteins

**Figure 7:**
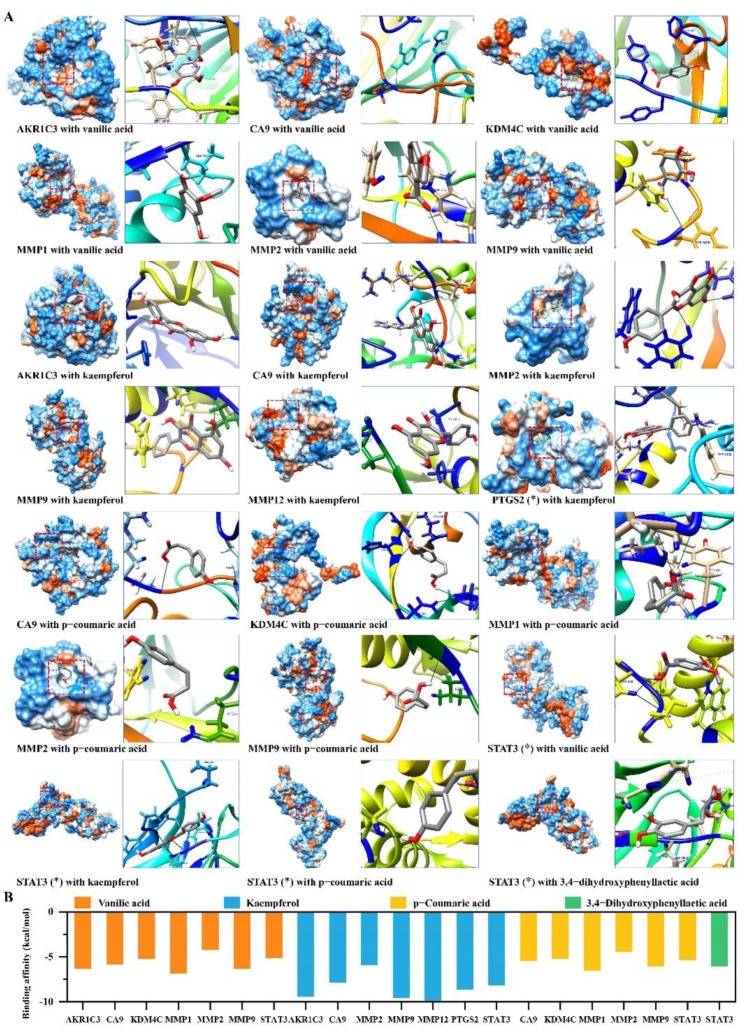
The main active components of SYF interact with candidate target proteins. (**A**) The binding action and binding site of the compounds to their targets. Amino acid residues that hydrogen bond to the compound were marked in blue. * Only part of the amino acid structure of the binding region of PTGS2 or STAT3 is displayed due to the molecular weight of the protein exceeding the calculation limit of the software. (**B**) The results of the binding affinity between the compounds and their targets.

The candidate active compounds, such as kaempferol, p-coumaric acid, vanillic acid, and 3,4-dihydroxyphenyllactic acid, were obtained from network pharmacology and enrichment analyses. The interactions between these compounds and the candidate target proteins were evaluated using molecular docking verification. Most of these compounds (Supplementary Table S3) tightly bound to the candidate proteins (Figure 7A). Most of the binding affinity of the interactions was less than −5 kcal/mol (Figure 7B and Supplementary Table S4). In particular, kaempferol bound more easily to proteins such as MMPs, AKR1C3, and STAT3 (Figure 7B).

### 2.6. SYF Extract Reduced Tumour Metastasis via STAT3 Signalling

**Figure 8:**
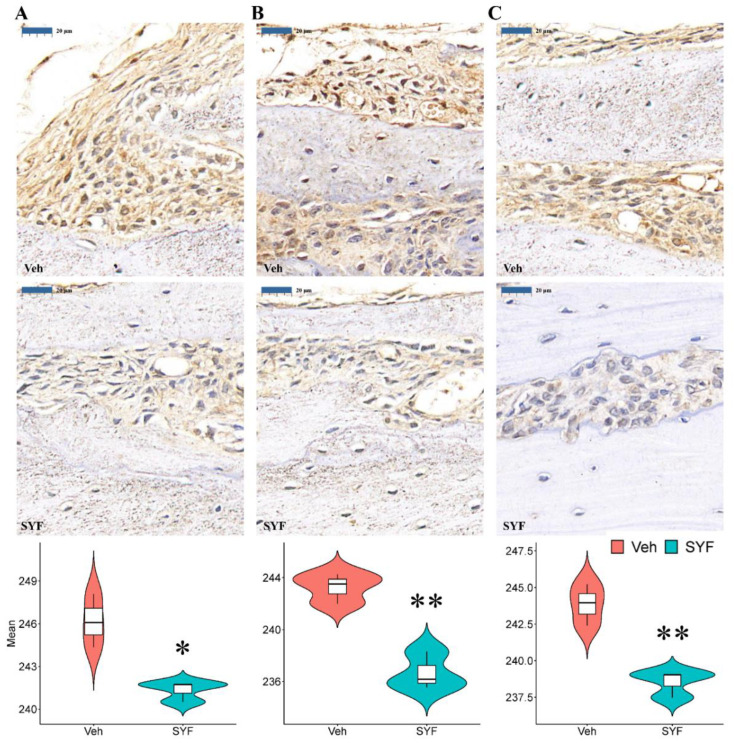
SYF reduced STAT3, MMP-2 and MMP-9 expression in bone metastases. Representative photographs and quantitative analysis of STAT3 (**A**), MMP-2 (**B**), and MMP-9 (**C**) IHC staining in bone metastases. * *p* < 0.05, ** *p* < 0.01 vs. Veh group.

To elucidate the molecular mechanism by which SYF inhibits breast cancer metastasis, IHC staining, qPCR, and Western blotting were used to determine the expression levels of tumour proliferation-, migration- and invasion-related genes. Interestingly, SYF extract significantly reduced STAT3, MMP-2, and MMP-9 protein expression in bone metastases (Figure 8).

**Figure 9:**
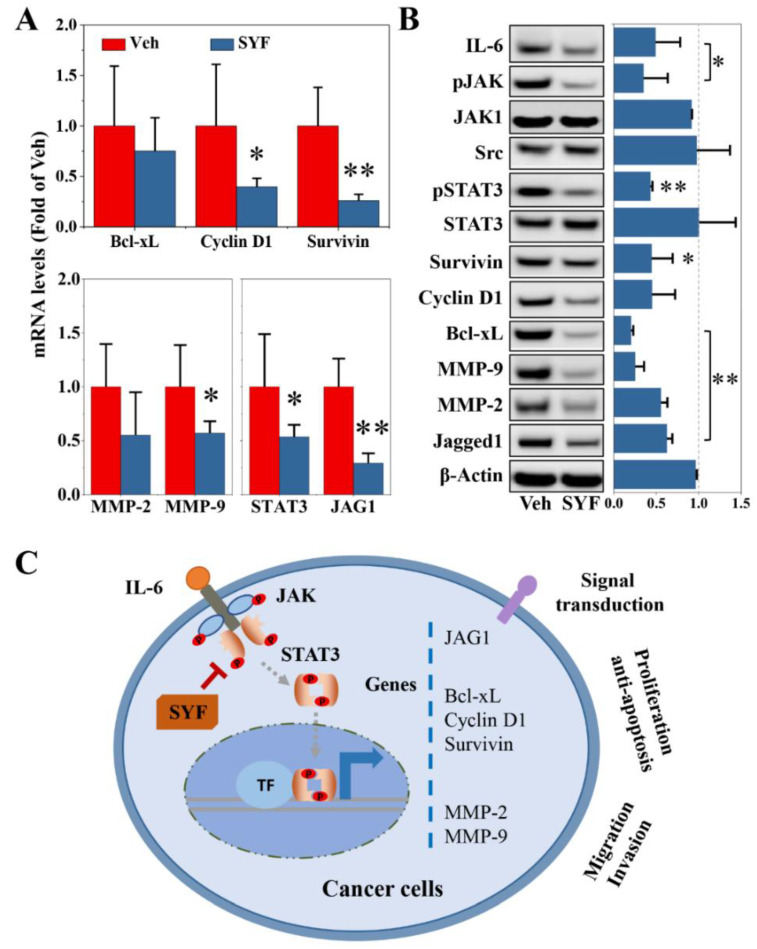
SYF reduced breast cancer cell metastasis by regulating cell cycle-, migration-, and invasion-related genes. (**A**) Effects of SYF on mRNA expression. Breast cancer cells were incubated with or without SYF (100 μg/mL) for 24 h and harvested for total RNA extraction. qPCR was used to determine mRNA expression (N = 3). (**B**) Representative pictures of the effect of SYF on candidate target proteins and relative quantitative results (fold of Veh). Breast cancer cells were incubated with or without SYF (100 μg/mL) for 24 h and harvested for total protein extraction. Western blotting was used to determine protein expression (N = 3). (**C**) Schematic diagram of the inhibitory mechanism of SYF in breast cancer. JAG1, Jagged1. * *p* < 0.05, ** *p* < 0.01 vs. Veh group.

Additionally, SYF significantly reduced the expression levels of cell proliferation- and apoptosis-related genes, such as cyclin D1 and survivin in MDA-MB-231 cells (Figure 9A). The mRNA expression of the migration- and invasion-related *MMP-2* and *MMP-9* were also inhibited by SYF treatment (Figure 9A). In addition, SYF reduced the STAT3 and Jagged1 mRNA levels (Figure 9A). Consistent results were confirmed in the Western blot analysis (Figure 9B). IL-6, survivin, Bcl-xL, MMPs, and Jagged1 were inhibited by SYF; the phosphorylation of JAK and STAT3 was also significantly inhibited by SYF (Figure 9B).

## 3. Discussion

We previously found that SYF significantly prolonged DFS in female patients with early-stage TNBC [12,13]. This study aimed to reveal the active components and molecular mechanisms of SYF against TNBC. We found that kaempferol, p-coumaric acid, 3,4-dihydroxyphenyllactic acid, and vanillic acid may be the active components of SYF. SYF extract inhibited TNBC cell metastasis by regulating cell proliferation, invasion, and migration via the JAK/STAT3 signalling pathway.

It is extremely challenging to study the active components of SYF, which contains nine Chinese herbal medicines, due to its numerous ingredients. We preliminarily identified four components through a network pharmacology approach with TCMSP database screening combined with HPLC-MS/MS analysis. Finally, 40 candidate targets of SYF in TNBC were identified by intersecting the targets of these compounds in the TTD and TNBC targets in the DisGeNET database using Venn diagram analysis. The results of the enrichment analysis and confirmatory experiments confirmed that STAT3 may be the most important key target, suggesting that STAT3 signalling may be the key pathway for SYF to inhibit TNBC cell metastasis. Furthermore, 3,4-dihydroxyphenyllactic acid, kaempferol, p-coumaric acid, and vanillic acid may be the active components of SYF. Previous studies showed that these potential active components exhibit antitumor effects. Kaempferol inhibits breast cancer cell proliferation, induces apoptosis, and suppresses cell migration by regulating the AKT, MEK, and PKC/MAPK pathways [14,15,16,17]. p-Coumaric acid induces cancer cell apoptosis by regulating nuclear damage and repair and apoptosis-related gene expression in MDA-MB-231 cells [18]. Vanillic acid was found to reduce tumour growth through the STAT3 and p38 MAPK signalling pathways in melanoma-cell-bearing mice [19]. Additionally, icariin inhibits osteoclast formation through the RANKL-mediated NF-κB/ERK pathway, indicating that it is a candidate therapeutic agent for breast cancer bone metastasis [20]. Danshensu (3,4-dihydroxyphenyllactic acid) inhibited cell migration and invasion via p38 signalling in human oral cancer [21]. Additionally, Danshensu inhibited MAOB activity and NF-κB signalling, and ultimately enhanced the radiation efficacy of non-small-cell lung cancer [22].

In addition to the abovementioned active ingredients, a variety of compounds with relatively high contents, which were identified by HPLC-MS/MS but not found to be enriched by network pharmacology analysis, exhibit antitumor effects. Icariin induces breast cancer cell apoptosis through the PI3K/AKT and SIRT6/NF-κB signalling pathways [23,24]. Icariin also induces apoptosis in tamoxifen-resistant breast cancer cells, suggesting that it may be used as an alternative treatment in cases with chemotherapeutic resistance [25]. 5-Hydroxymethylfuraldehyde inhibits cell migration and invasion through ion conductance regulated by aquaporin-1 in TNBC cells [26]. Magnoflorine induces cancer cell apoptosis and autophagy by regulating PI3K/AKT/mTOR and p38 MAPK signalling [27,28]. Furanodiene reduces cancer cell adhesion, migration, and invasion through the PI3K/AKT signalling pathway in MDA-MB-231 cells [29]. In addition, magnoflorine enhances the anticancer effects of doxorubicin by regulating FAK and AKT signalling [30,31]. Protocatechualdehyde inhibits cell proliferation and induces apoptosis by regulating GSK3β- and NF-κB-mediated proteasomal degradation in TNBC cells [32]. The results of this study further confirmed that SYF can inhibit bone metastasis of TNBC cells, consistent with a previous study suggesting that icariin may play a key role in regulating the microenvironment of bone metastasis [20]. These components extracted from SYF not only inhibit the proliferation, invasion, and metastasis of breast cancer cells but also ultimately inhibit bone metastasis of TNBC cells by regulating bone metabolism. We are preparing these components and continuing to study their inhibitory effects alone and in combination on TNBC. We expect to identify single components or compatible combinations with better purity and better efficacy.

As a significant type of protease, MMPs play key roles in many biological processes, including cancer cell invasion, metastasis, and angiogenesis [33]. MMPs were associated with lymph node metastasis in patients with early cervical cancers, indicating MMPs may be key regulators of early cancer metastasis [34]. MMPs can cleave many extracellular matrix (ECM) proteins to regulate ECM remodelling [35]. ECM remodelling and degradation is required in the early steps of the metastatic cascade, such as invasion, tumour intravasation, and extravasation [36]. Exosomal MMPs secreted by tumour-associated macrophages or other cells promoted cancer metastasis by facilitating the EMT process [37,38]. Clinical osteoblastic bone metastases have higher levels of MMP-3 compared to other visceral metastases [39]. Inhibition of MMP-9 expression by microRNA-429 significantly inhibited bone metastasis of breast cancer, which suggested that MMPs may become therapeutic targets for cancer cell metastasis [40]. We found that SYF extract significantly inhibited the expression of MMPs in breast cancer cells, suggesting that SYF can effectively inhibit the metastasis of breast cancer. The results of the present study help to explain our previous clinical finding that additional DFS benefits were obtained with SYF plus chemotherapy compared with chemotherapy alone [13]. Our previous exploratory subgroup analysis showed that the benefit of SYF in patients with node-negative breast cancer was significantly higher than that in patients with node-positive breast cancer, which seemed to be achieved by SYF through the inhibition of MMPs to prevent breast cancer cell metastasis [13]. This finding further proves the effectiveness of MMP inhibitors in the treatment of breast cancer metastasis.

STAT3 is overexpressed and persistently activated in TNBC and plays a key role in tumour cell proliferation, migration, invasion, metastasis, and immune evasion [41,42]. STAT3 is involved in cell survival, cell proliferation, cell cycle progression, apoptosis resistance, migration, invasion, angiogenesis, and chemotherapeutic resistance by regulating the expression of its downstream target genes [43]. After upstream cytokines (IL-6, etc.) or growth factors (EGF, FGF, VEGF, etc.) bind to cell surface receptors, STAT3 is phosphorylated and activated by JAK, Src, etc. Activated STAT3 further binds to target DNA molecules and its coactivators and induces the transcription of its downstream target genes, such as cyclin D, c-myc, bcl-2, MMPs, Jagged1, and EMT-related genes [43,44,45]. Our network pharmacology and bioinformatic enrichment analyses showed that STAT3 may be the most important key target, suggesting that STAT3 signalling may be the key pathway for SYF to inhibit TNBC cell metastasis. We subsequently found that SYF extract inhibits the STAT3 signalling pathway. STAT3 downstream genes, such as cyclin D, bcl-xL, MMP2, MMP9, and Jagged1, were also inhibited by SYF extract, consistent with the enrichment analysis results. The results of this study indicated that SYF exerts its anticancer effect by inhibiting the STAT3 signalling pathway and then regulating its downstream genes, such as MMP-2 and MMP-9. In fact, a considerable number of STAT3-targeted therapies have been successfully developed and demonstrated efficacy in TNBC preclinical studies [46,47,48]. Some STAT3 inhibitors have even entered clinical trials, including TNBC [49]. In addition, STAT3 also plays a key role in the immune system. STAT3 inhibitors not only suppress tumour cells but also enhance immune cell responses [50,51]. Therefore, STAT3 inhibitors are a promising target for TNBC prevention and treatment.

## 4. Materials and Methods

### 4.1. Materials and Instruments

DMEM (Gibco, Grand Island, NY, USA), antibiotic–antimycotic solution (Gibco, Grand Island, NY, USA), foetal bovine serum (FBS) (Gibco, Grand Island, NY, USA), SYBR Green Master Mix (Takara Bio, Takara, Japan), PCR primers (Solarbio Life, Beijing, China), an RNase inhibitor (Sangon Biotech, Shanghai, China), Transwell chambers (Corning, Corning, NY, USA), Matrigel matrix (Corning, Corning, NY, USA), paclitaxel (Aladdin, Shanghai, China), and chromatography-grade acetonitrile (Merck, Darmstadt, Germany) were used. Antibodies specific for Bcl-xL (ab32370), cyclin D1 (ab134175), Jagged1 (ab109536), matrix metallopeptidase 2 (MMP-2) (ab86607), MMP-9 (ab137867), signal transducer and activator of transcription 3 (STAT3) (ab68153), phosphor-STAT3 (ab267373), survivin (ab76424), β-Actin (ab8226), horseradish peroxidase (HRP)-conjugated goat anti-mouse IgG (ab205719), and goat anti-rabbit IgG (ab205718) were purchased from Abcam (Cambridge, England). Antibodies specific for Src (#2109), phospho-Jak family (#32901), Jak1 (#3332), and IL-6 (#12153) were obtained from Cell Signaling Technology (Danvers, MA, USA). All other chemicals were obtained from Shanghai Sinopharm (Shanghai, China), unless otherwise indicated.

### 4.2. Traditional Chinese Medicines and Process for Extracting SYF Extract

SYF is composed of nine traditional Chinese medicines in the following quantities: *Codonopsis pilosula* Nannf. (Chinese name: Dangshen, 12 g), *Atractylodes macrocephala* Koidz. (Chinese name: Baizhu, 12 g), *Poria cocos* (Schw.) Wolf. (Chinese name: Fuling, 12 g), *Salviae Chinensis* Herba (Chinese name: Shijianchuan, 30 g), *Curcuma phaeocaulis* Valeton (Chinese name: Ezhu, 30 g), *Epimedium brevicornu* Maxim. (Chinese name: Yinyanghuo, 15 g), *Solanum nigrum* Linn. (Chinese name: Longkui, 30 g), *Scutellariae Barbatae* Herba (Chinese name: Banzhilian, 30 g), and *Prunella vulgaris* Linn. (Chinese name: Xiakucao, 9 g). The total daily dose was 180 g. All these medicines were obtained from Shanghai Kangqiao Chinese Medicine Tablet Co., Ltd. (Shanghai, China). The purchased Chinese medicines were identified as authentic medicinal materials by Chinese medicine experts in the pharmacy department of our hospital.

The process for extracting SYF extract was carried out according to our previous study [52]. In brief, the medicinal materials were initially extracted with a 10-fold volume of water, and the residue was continuously decocted with an 8-fold volume of water. The two filtrates were combined and concentrated to yield the extract. The samples were stored in an airtight container in a cool location after freeze-drying.

### 4.3. HPLC-MS/MS Analysis

An Agilent 1260 Series high-performance liquid chromatograph (Agilent Technologies, Santa Clara, CA, USA) coupled with an Agilent 6530 quadrupole/time-of-flight (Q-TOF) mass spectrometer (Agilent Technologies, Santa Clara, CA, USA) with a dual jet stream ion source was used to identify the compounds in SYF extract. HPLC-MS/MS analysis was performed according to a previous study [53] with the following main parameters: drying gas temperature, 300 °C; flow rate, 6.0 L/min; nebulizer pressure, 45 psi; sheath gas temperature, 300 °C; capillary voltage, 4000 V; nozzle voltage, 500 V; skimmer voltage, 65 V; capillary outlet voltage, 100 V; collision energy in both positive and negative ion modes, 45 V; spectrum acquisition frequency, 2 per second (primary mass spectrum) and 1 per second (secondary mass spectrum). Agilent Technologies MassHunter Workstation Q-TOF Acquisition Software (version B.05.01, Agilent Technologies, Santa Clara, CA, USA) was used for data acquisition. Agilent Technologies MassHunter Workstation Quantitative Analysis Software (version B.06.00, Agilent Technologies, Santa Clara, CA, USA) was used for qualitative data analysis.

### 4.4. Cells and Cell Culture

The firefly luciferase-labelled human TNBC cell line MDA-MB-231 was purchased from the Shanghai Institute of Cell Biology (SICB, Shanghai, China), Chinese Academy of Sciences (Shanghai, China). According to the SICB *Culture Recommendation Manual*, breast cancer cells were cultured in DMEM containing 10% FBS at 37 °C, with 5% CO_2_ in an incubator (Thermo Fisher, MA, USA).

### 4.5. Identification of Candidate Targets of SYF in TNBC

TCMSP (version 2.3, accessed on 2 February 2022, https://www.tcmsp-e.com/) was used to search and analyse the chemical constituents of traditional Chinese medicines in SYF mentioned above. After removing the repeated components, the candidate components were identified by screening with the criterion of oral availability greater than 30%. The candidate compounds were initially identified by intersecting the compounds detected by HPLC-MS/MS with those identified in the TCMSP database. The targets of these compounds, which are shown in Supplementary Table S2, were then searched using the TTD (accessed on 19 February 2022, http://db.idrblab.net/ttd/). These targets were then intersected with TNBC targets in the DisGeNET database (version 7.0, accessed on 19 February 2022, https://www.disgenet.org/home/). Finally, the candidate anti-TNBC target of SYF was identified.

### 4.6. Bioinformatic Enrichment Analyses

Enrichment analyses were performed according to previous work [54]. In brief, the candidate targets identified as described above were subjected to enrichment analysis with REACTOME (released version 79, accessed on 13 March 2022, https://reactome.org/). In addition, protein–protein interaction (PPI) analysis and gene-miRNA, transcription factor (TF)-gene, and TF-miRNA network analyses were performed using NetworkAnalyst (version 3.0, accessed on 15 March 2022, https://www.networkanalyst.ca/).

### 4.7. Cell Proliferation Assay

The cell proliferation assay was performed using a Celigo imaging cytometer according to our previous study, with slight modifications [52]. In brief, cells were cultured at a density of 2000 cells/well in a 96-well plate. The cell growth curve was drawn to indicate cell proliferation after 5 days of continuous image recording with the Celigo system. Cell numbers were normalized to the cell numbers on the first day.

### 4.8. Transwell Invasion and Migration Assays

The cell invasion and migration assays were performed using Transwell chambers containing membranes coated with or without Matrigel matrix according to our previous study, with slight modifications [52]. In brief, cells were seeded in the upper chamber containing FBS-free medium. The medium in the lower chamber contained 20% FBS. After incubation with or without SYF for the indicated times, the invaded cells were stained with Giemsa and counted under an inverted light microscope. The cell seeding density in a 24-well plate was 5000 cells/well for the migration assay and 10,000 cells/well for the invasion assay. Matrigel matrix was used in the invasion assay and was omitted in the migration assay.

### 4.9. Wound-Healing Migration Assay

The wound-healing assay was carried out according to previous work, with slight modifications [52]. In brief, cells were cultured at a density of 50,000 cells/well in 96-well plates. At approximately 90% confluence, the cell layer was scratched using a wounding replicator, and the medium containing 10% FBS was replaced with an FBS-free medium. The cells were incubated and photographed with a light microscope at the indicated times.

### 4.10. Quantitative PCR (qPCR)

qPCR was carried out in a LightCycler 480 system (Roche Diagnostics, Indianapolis, IN, USA) with SYBR Green Master Mix (Roche Diagnostics, Indianapolis, IN, USA), as described in our previous work [52]. Briefly, total RNA was extracted using QIAGEN RNeasy Mini Kit according to the instructions. RNA was then treated with DNase I to remove residual genomic DNA. The qualified RNA (the ratio of 260 nm/280 nm absorbance value was between 1.9 and 2.1) was used for the subsequent PCR reaction. Oligo(dT) was used for reverse transcription into cDNA. PCR amplification was performed using the cDNA as a template. The primers used are listed in Supplementary Table S5. Gene expression levels were normalized to those of GAPDH. The relative quantitative analysis of mRNA expression was performed using the 2^-ΔΔCt^ method.

### 4.11. Western Blotting (WB)

Western blotting was performed using a Mini-PROTEAN Tetra cell system (Bio-Rad, Hercules, CA, USA) as described in previous work [52]. The total amount of protein loaded was 20 μg. Total protein was separated on an 10% SDS-page gel and then transferred to a PVDF membrane. After blocking the unbound sites on the PVDF membrane with BSA, the primary antibody and the secondary antibody (HRP-labelled) were incubated sequentially. Subsequent addition of HRP substrate produced a developing reaction. The final protein image was photographed. The dilution factor of the antibody was carried out according to the manufacturer’s instructions. Relative quantitative analysis of protein expression was carried out with ImageJ (Version: 1.8.0, National Institutes of Health, Bethesda, MD, USA).

### 4.12. Animals and Xenograft Model Establishment

Five-week-old female nude (BALB/c nu/nu) mice were housed in a temperature-controlled (24 ± 2 °C) specific-pathogen-free (SPF) environment on a regular 12 h light/dark cycle. All animals had free access to water and food throughout the study period. All animal experiments were performed according to the national regulations for animal experimentation and approved by the Institutional Animal Care and Use Committee of the First Affiliated Hospital of Zhejiang Chinese Medical University. The approved number was 2020-KL-168-01 (approved at 29/01/2021).

Tumours were generated in mice as described in our previous work [52]. In brief, firefly luciferase-labelled MDA-MB-231 cells (0.1 mL/mouse, 1 × 10^7^ cells/mL) were injected into the left ventricle of mice. All tumour-bearing mice were randomly divided into four groups (*n* = 6 mice/group). Mice in group one received SYF extract (30 g of crude drug/kg body weight, p.o., q.d.); mice in group two received PTX (10 mg/kg) via intraperitoneal injection (i.p.) twice a week; mice in group three received PTX (10 mg/kg, i.p., twice a week) plus SYF extract (30 g of crude drug/kg, p.o., q.d.); mice in the last group were administered a vehicle (10 mL/kg). The treatments continued for 4 weeks. The dose of SYF was converted from the equivalent dose according to the clinical dosage used in TNBC patients (180 g crude drug/day) according to our previous studies [12,13]. This equivalent dose conversion is also according to the FDA recommendations, and the dose of mice is about 12-fold that of human [55]. After mice were anaesthetized with sodium pentobarbital (50 mg/kg), in vivo imaging was used to monitor the intensity of the bioluminescence signal weekly to evaluate tumour cell proliferation. At the end of the experiment, mice were sacrificed after anaesthesia with sodium pentobarbital (50 mg/kg), and lung metastases and bone metastases were removed and fixed with formalin for pathological analysis.

### 4.13. Haematoxylin-Eosin (HE), Tartrate-Resistant Acid Phosphatase (TRAP), and Giemsa Staining

HE, TRAP, and Giemsa staining were performed as described in our previous work [52]. The migration and invasion of cells were recorded with a Celigo imaging cytometer (Nexcelom Bioscience, Lawrence, MA, USA). Photographs of bone metastases and lung metastases were scanned with Pannoramic DESK P-MIDI, P250 (Version 2.4, 3D HISTECH Ltd., Budapest, Hungary).

### 4.14. Immunohistochemical (IHC) Staining and Semiquantitative Analysis

IHC staining was carried out as described in a previous study [56]. In brief, antigen retrieval was performed on paraffin sections using EDTA antigen-retrieval solution. The sections were then incubated with hydrogen peroxide solution, followed by blocking with BSA to block endogenous peroxidase. The primary and secondary antibodies (HRP-conjugated) were then incubated sequentially and diluted according to the antibody instructions. After elution, the colour reaction was carried out with DAB chromogenic solution. Nuclei were then counterstained with haematoxylin. After the final closure, the photos were scanned with a Pannoramic DESK P-MIDI, P250 (Version 2.4, 3D HISTECH Ltd., Budapest, Hungary). Quantitative analysis of histological staining was performed using ImageJ (version: 1.8.0, National Institutes of Health, Bethesda, MD, USA).

### 4.15. Molecular Docking

The molecular docking analysis was conducted with UCSF Chimera (Version 1.16, University of California, San Francisco, CA, USA) and Autodock Vina (Version 1.12, Scripps Research Institute, La Jolla, CA, USA), as described in a previous work [57]. In brief, the protein PDB structure, which was obtained from the Protein Data Bank (PDB) at https://www.rcsb.org/ (accessed on 30 August 2022), was loaded using Chimera, and nonprotein components were removed. Proteins were minimized, which included the addition of hydrogen and charges. Then, the candidate compound was imported. Binding analysis was analysed by Autodock Vina. The analysis was performed in the range of compounds <2.5 angstroms. At the same time, the binding affinity was calculated. The candidate compounds and their target proteins were obtained using intersection analysis between the targets identified using Materials and Methods 2.5 and the results of the Swiss Target Prediction (http://www.swisstargetprediction.ch/, accessed on 29 August 2022). The potential target protein access IDs of these compounds are referred to in Supplementary Table S4.

### 4.16. Statistical Analysis

All results are presented as the mean ± standard deviation values. A one-sample Student’s *t*-test was used to evaluate differences between the SYF group and vehicle group. Statistical significance was set at *p* < 0.05.

## 5. Conclusions

The results of the present study indicate that SYF regulates the expression of genes involved in cell proliferation, migration, and invasion by regulating the JAK/STAT3 signalling pathway and finally enhances the therapeutic efficacy of paclitaxel in triple-negative breast cancer (Figure 9C). The results of this study directly confirm that SYF inhibited cell proliferation, migration, and invasion for the first time in TNBC cells. These inhibitory results elucidate the molecular mechanism by which SYF reduces TNBC recurrence and metastasis and ultimately significantly prolongs the DFS in TNBC patients [12,13]. The present study not only clarifies the potential active ingredients of SYF and preliminary mechanism by which SYF inhibits TNBC cell metastasis, but also lays an experimental foundation for the continued clinical development of SYF targeting the JAK/STAT3 pathway.

## Data Availability

All data sets obtained during and/or analysed during the current study are available from the corresponding author upon request.

## Supplementary Materials

The following supporting information can be downloaded at: https://www.mdpi.com/article/10.3390/ph16010009/s1, Table S1: Main compounds determined by HPLC-MS/MS; Table S2: Candidate targets; Table S3: Candidate active ingredient of SYF extracts; Table S4: The main parameters and results of the molecular docking; Table S5: RT-qPCR primer information.

## Abbreviations

BLI: bioluminescence imaging; ER, oestrogen receptor; HER2, human epidermal growth factor receptor 2; HPLC-MS/MS, high-performance liquid chromatography–tandem mass spectrometry; PPI, protein–protein interaction; PR, progesterone receptor; qPCR, quantitative PCR; STAT3, signal transducer and activator of transcription 3; SYF, Sanyin formula; TCMSP, Traditional Chinese Medicine Systems Pharmacology Database and Analysis Platform; TNBC, triple-negative breast cancer.
