# Supplementary material for: Sanyin Formula Enhances the Therapeutic Efficacy of Paclitaxel in Triple-Negative Breast Cancer Metastases through the JAK/STAT3 Pathway in Mice
Source: Pharmaceuticals (Basel). 2022 Dec 22;16(1):9. doi: 10.3390/ph16010009 (PMC9867389; doi:10.3390/ph16010009)
Supplement: Supplementary file 1 [file pharmaceuticals-16-00009-s001.zip › pharmaceuticals-2073611-supplementary.pdf]

Supplementary Table S1. Main compounds determined by HPLC-MS/MS.

No.	Retention time (min)	Identification	Molecular formula	Ion mode	Source
1	1.207	Malic acid	C4H6O5	$[M-H]^-$	*Prunella vulgaris* Linn.
2	2.02	Gallic acid	C7H6O5	$[M-H]^-$	*Poria cocos* (Schw.) Wolf.
3	2.291	Quinic acid	C7H12O6	$[M-H]^-$	*Atractylodes macrocephala* Koidz.
4	2.9	Codonopsine	C14H21NO4	$[M+H]^+$	*Codonopsis pilosula* Nannf.
5	2.955	Danshensu	C9H10O5	$[M-H]^-$	*Prunella vulgaris* Linn.
6	2.901	Vanillic acid	C8H8O4	$[M-H]^-$	*Codonopsis pilosula* Nannf.
7	3.307	Ethyl gallate	C9H10O5	$[M-H]^-$	*Poria cocos* (Schw.) Wolf.
8	3.327	Danshensu or its isomer	C9H10O5	$[M-H]^-$	*Prunella vulgaris* Linn.
9	4.052	3,4-Dihydroxybenzoic acid	C7H6O4	$[M-H]^-$	*Prunella vulgaris* Linn.
10	4.119	5-Hydroxymethylfuraldehyde	C6H6O3	$[M+H]^+$	*Codonopsis pilosula* Nannf.
11	5.136	Chlorogenic acid	C16H18O9	$[M-H]^-$	*Codonopsis pilosula* Nannf.
12	5.887	4-Hydroxybenzoic acid	C7H6O3	$[M-H]^-$	*Prunella vulgaris* Linn.
13	5.949	Protocatechualdehyde	C7H6O3	$[M+H]^+$	*Salviae chinensis* Herba
14	7.439	Chlorogenic acid or its isomer	C16H18O9	$[M-H]^-$	*Codonopsis pilosula* Nannf.
15	8.116	Caffeic acid	C9H8O4	$[M-H]^-$	*Prunella vulgaris* Linn.
16	8.725	Magnoflorine	C20H23O4N	$[M+H]^+$	*Epimedium brevicornu* Maxim.
17	9.132	3-O-p-coumaroylquinic acid	C16H18O8	$[M-H]^-$	*Epimedium brevicornu* Maxim.
18	9.2	p-Coumaric acid	C9H8O3	$[M-H]^-$	*Prunella vulgaris* Linn.
19	9.34	p-hydroxycinnamic acid	C9H8O3	$[M-H]^-$	*Epimedium brevicornu* Maxim.
20	10.081	Isomyricitrin	C21H20O13	$[M-H]^-$	*Epimedium brevicornu* Maxim.
21	10.35	Curcumenone or its isomer	C15H22O2	$[M+H]^+$	*Curcuma phaeocaulis* Valeton
22	10.825	Codonopyrrolidium A	C19H28NO5	$[M+H]^+$	*Codonopsis pilosula* Nannf.
23	11.028	Scopoletin	C10H8O4	$[M+H]^+$	*Solanum nigrum* Linn.
24	11.435	Rutin	C27H30O16	$[M-H]^-$	*Prunella vulgaris* Linn.

25	11.638	Carthamidin-7-O-glucuronide	C21H20O12	[M-H]-	*Scutellariae barbatae* Herba
26	11.748	Hyperoside	C21H20O12	[M-H]-	*Prunella vulgaris* Linn.
27	11.841	Perlolyrine	C16H12N2O2	[M+H]+	*Codonopsis pilosula* Nannf.
28	11.909	Apigenin-7-O-glucuronide	C21H18O11	[M-H]-	*Scutellariae barbatae* Herba
29	12.044	Luteolin-7-O-glucuronide	C21H18O12	[M+H]+	*Scutellariae barbatae* Herba
30	12.179	Apigenin	C15H10O5	[M+H]+	*Scutellariae barbatae* Herba
31	12.201	Emodin	C15H10O5	[M+H]+	*Epimedium brevicornu* Maxim.
32	12.248	Isocarthamidin-7-O-glucuronide	C21H20O12	[M-H]-	*Scutellariae barbatae* Herba
33	12.568	Scutellarein	C15H10O6	[M+H]+	*Scutellariae barbatae* Herba
34	12.586	Luteoloside	C21H20O11	[M+H]+	*Prunella vulgaris* Linn.
35	12.611	Luteolin-β-D-glucoside	C21H20O11	[M+H]+	*Scutellariae barbatae* Herba
36	13.196	Azelaic acid or its isomer	C9H16O4	[M-H]-	*Codonopsis pilosula* Nannf.
37	13.223	Azelaic acid	C9H16O4	[M-H]-	*Codonopsis pilosula* Nannf.
38	13.331	Curcumenone or its isomer	C15H22O2	[M+H]+	*Curcuma phaeocaulis* Valeton
39	13.467	4'-Hydroxy-wogonin-7-O-Glucuronide	C22H20O12	[M-H]-	*Scutellariae barbatae* Herba
40	13.67	Rosmarinic acid	C18H16O8	[M-H]-	*Prunella vulgaris* Linn.
41	13.72	Rosmarinic acid	C18H16O8	[M-H]-	*Salviae chinensis* Herba
42	13.874	Lobetyolin	C20H28O8	[M-H]-	*Codonopsis pilosula* Nannf.
43	14.414	Atractylenolide-2	C15H18O2	[M+H]+	*Atractylodes macrocephala* Koidz.
44	14.55	Solaonine	C45H73NO16	[M+H]+	*Solanum nigrum* Linn.
45	14.685	Scutellarin	C21H18O12	[M+H]+	*Scutellariae barbatae* Herba
46	14.753	Luteolin	C15H10O6	[M+H]+	*Scutellariae barbatae* Herba
47	14.956	Solamargine	C45H73NO15	[M+H]+	*Solanum nigrum* Linn.
48	15.227	Furanodiene or its isomer	C15H20O	[M+H]+	*Curcuma phaeocaulis* Valeton
49	15.566	Dehydrocostus lactone or its isomer	C15H24O	[M+H]+	*Curcuma phaeocaulis* Valeton
50	16.244	Kaempferol	C15H10O6	[M-H]-	*Salviae chinensis* Herba

51	16.311	Epimedin B	C38H48O19	$[M+H]^+$	*Epimedium brevicornu* Maxim.
52	16.446	Tetradecylcitric acid	C20H36O7	$[M+H]^+$	*Atractylodes macrocephala* Koidz.
53	16.514	Epmedin C	C39H50O19	$[M+H]^+$	*Epimedium brevicornu* Maxim.
54	16.853	Icariin	C33H40O15	$[M+H]^+$	*Epimedium brevicornu* Maxim.
55	16.988	Dehydrocostus lactone	C15H24O	$[M+H]^+$	*Curcuma phaeocaulis* Valeton
56	17.936	Curdione	C15H24O2	$[M+H]^+$	*Curcuma phaeocaulis* Valeton
57	18.072	anhydroicaritin-3-O-rhamnoside (1-2) furan acid-7-O-glucoside	C39H48O19	$[M+H]^+$	*Epimedium brevicornu* Maxim.
58	18.141	7-Hydroxycoumarin	C9H6O3	$[M-H]^-$	*Prunella vulgaris* Linn.
59	18.208	4'-Hydroxy-wogonin	C16H12O6	$[M+H]^+$	*Scutellariae barbatae* Herba
60	18.275	Apigenin or its isomer	C15H10O5	$[M+H]^+$	*Scutellariae barbatae* Herba
61	18.327	Emodin or its isomer	C15H10O5	$[M+H]^+$	*Epimedium brevicornu* Maxim.
62	18.344	9,12,13-trihydroxy-10,15-octadecadienoic acid or its isomer	C18H32O5	$[M-H]^-$	*Codonopsis pilosula* Nannf.
63	18.546	Scutevulin	C16H12O6	$[M+H]^+$	*Scutellariae barbatae* Herba
64	19.291	Solasodine	C27H43NO2	$[M+H]^+$	*Solanum nigrum* Linn.
65	19.359	Curcumenone or its isomer	C15H22O2	$[M+H]^+$	*Curcuma phaeocaulis* Valeton
66	19.402	Furanodiene or its isomer	C15H20O	$[M+H]^+$	*Curcuma phaeocaulis* Valeton
67	19.459	9,12,13-trihydroxy-10-octadecenoic acid	C18H34O5	$[M-H]^-$	*Codonopsis pilosula* Nannf.
68	19.563	9,12,13-trihydroxy-10,15-Octadecadienoic acid or its isomer	C18H32O5	$[M-H]^-$	*Codonopsis pilosula* Nannf.
69	19.97	9,12,13-trihydroxy-10,15-Octadecadienoic acid or its isomer	C18H32O5	$[M-H]^-$	*Codonopsis pilosula* Nannf.
70	20.172	Epimedin L	C45H56O23	$[M+H]^+$	*Epimedium brevicornu* Maxim.
71	20.213	Epimedin L	C45H56O23	$[M+H]^+$	*Epimedium brevicornu* Maxim.
72	20.376	9,12,13-trihydroxy-10,15-Octadecadienoic acid or its	C18H32O5	$[M-H]^-$	*Codonopsis pilosula* Nannf.

		isomer			
73	20.443	Icariside A	$C_{26}H_{28}O_{10}$	$[M+H]^+$	*Epimedium brevicornu* Maxim.
74	20.51	Curcumenone or its isomer	$C_{15}H_{22}O_2$	$[M+H]^+$	*Curcuma phaeocaulis* Valeton
75	20.917	Atractylenolide-1	$C_{15}H_{18}O_2$	$[M+H]^+$	*Atractylodes macrocephala* Koidz.
76	21.391	Curcumenone isomer	$C_{15}H_{22}O_2$	$[M+H]^+$	*Curcuma phaeocaulis* Valeton
77	21.459	Isoasterolide A	$C_{15}H_{20}O_2$	$[M+H]^+$	*Atractylodes macrocephala* Koidz.
78	21.594	Skullcapflavone II	$C_{19}H_{18}O_8$	$[M+H]^+$	*Prunella vulgaris* Linn.
79	21.622	Methyl rosmarinate	$C_{19}H_{18}O_8$	$[M+H]^+$	*Salviae chinensis* Herba
80	22.339	Sagittatoside B	$C_{32}H_{38}O_{14}$	$[M+H]^+$	*Epimedium brevicornu* Maxim.
81	22.475	2″-O-rhamnosylikariside II	$C_{33}H_{40}O_{14}$	$[M+H]^+$	*Epimedium brevicornu* Maxim.
82	22.542	Curcumenone or its isomer	$C_{15}H_{22}O_2$	$[M+H]^+$	*Curcuma phaeocaulis* Valeton
83	22.813	2″-O-rhamnosylikariside II or its isomer	$C_{33}H_{40}O_{14}$	$[M+H]^+$	*Epimedium brevicornu* Maxim.
84	23.83	Baohuoside I	$C_{27}H_{30}O_{10}$	$[M-H]^-$	*Epimedium brevicornu* Maxim.
85	23.965	Curcumenone	$C_{15}H_{22}O_2$	$[M+H]^+$	*Curcuma phaeocaulis* Valeton
86	24.021	Furanodiene or its isomer	$C_{15}H_{20}O$	$[M+H]^+$	*Curcuma phaeocaulis* Valeton
87	25.048	Protocatechualdehyde or its isomer	$C_7H_6O_3$	$[M+H]^+$	*Salviae chinensis* Herba
88	26.472	9,10-dihydroxy-12-octadecenoic acid or its isomer	$C_{18}H_{34}O_4$	$[M-H]^-$	*Codonopsis pilosula* Nannf.
89	26.946	9,10-dihydroxy-12-octadecenoic acid	$C_{18}H_{34}O_4$	$[M-H]^-$	*Codonopsis pilosula* Nannf.
90	27.013	Furanodiene or its isomer	$C_{15}H_{20}O$	$[M+H]^+$	*Curcuma phaeocaulis* Valeton
91	27.216	Gweicurculactone	$C_{15}H_{16}O_2$	$[M+H]^+$	*Curcuma phaeocaulis* Valeton
92	27.487	3-O-(4-acetoxy) rhamnose-2-o-(m-diacetoxy) grape Sugar icariin or its isomers	$C_{39}H_{46}O_{18}$	$[M+H]^+$	*Epimedium brevicornu* Maxim.
93	27.758	3-O-(4-acetoxy) rhamnose-2-o - (m-diacetoxy) grape Sugar icariin or its isomers	$C_{39}H_{46}O_{18}$	$[M+H]^+$	*Epimedium brevicornu* Maxim.
94	29.248	Mannopyranoside	$C_{26}H_{48}O_{10}$	$[M+H]^+$	*Atractylodes macrocephala* Koidz.
95	29.654	Furanodiene	$C_{15}H_{20}O$	$[M+H]^+$	*Curcuma phaeocaulis* Valeton

96	31.214	9-hydroxy-10,12-octadecadienoic acid	C18H32O3	$[M-H]^-$	*Codonopsis pilosula* Nannf.
97	32.025	Curcumin	C21H20O6	$[M+H]^+$	*Curcuma phaeocaulis* Valeton
98	32.112	Icaritin	C21H20O6	$[M+H]^+$	*Epimedium brevicornu* Maxim.
99	32.973	Germacrone	C15H22O	$[M+H]^+$	*Curcuma phaeocaulis* Valeton

Supplementary Table S2. Candidate targets.

No.	ID	Name	No.	ID	Name
1	AKR1C3	Aldo-Keto Reductase Family 1 Member C3	21	LPAR3	Lysophosphatidic Acid Receptor 3
2	AR	Androgen Receptor	22	MAOB	Monoamine Oxidase B
3	CA9	Carbonic Anhydrase 9	23	MMP1	Matrix Metallopeptidase 1
4	CXCR2	C-X-C Motif Chemokine Receptor 2	24	MMP2	Matrix Metallopeptidase 2
5	EDNRA	Endothelin Receptor Type A	25	MMP7	Matrix Metallopeptidase 7
6	FABP4	Fatty Acid Binding Protein 4	26	MMP9	Matrix Metallopeptidase 9
7	FABP5	Fatty Acid Binding Protein 5	27	MMP12	Matrix Metallopeptidase 12
8	FOLH1	Folate Hydrolase 1	28	NR1I2	Nuclear Receptor Subfamily 1 Group I Member 2
9	GABRA3	Gamma-Aminobutyric Acid Type A Receptor Subunit Alpha3	29	PDGFRA	Platelet Derived Growth Factor Receptor Alpha
10	GRM1	Glutamate Metabotropic Receptor 1	30	PDGFRB	Platelet Derived Growth Factor Receptor Beta
11	GSR	Glutathione-Disulfide Reductase	31	PIN1	Peptidylprolyl Cis/Trans Isomerase, NIMA-Interacting 1
12	HDAC1	Histone Deacetylase 1	32	PPARG	Peroxisome Proliferator Activated Receptor Gamma
13	HDAC6	Histone Deacetylase 6	33	PTGS2	Prostaglandin-Endoperoxide Synthase 2
14	HDAC8	Histone Deacetylase 8	34	SLC25A1	Solute Carrier Family 25 Member 1
15	ITGB3	Integrin Subunit Beta 3	35	SLC6A3	Solute Carrier Family 6 Member 3
16	KDM1A	Lysine Demethylase 1A	36	SRD5A1	Steroid 5 Alpha-Reductase 1
17	KDM4C	Lysine Demethylase 4C	37	STAT3	Signal Transducer And Activator Of Transcription 3
18	KIF11	Kinesin Family Member 11	38	TACR1	Tachykinin Receptor 1
19	LDHA	Lactate Dehydrogenase A	39	THRB	Thyroid Hormone Receptor Beta
20	LDHB	Lactate Dehydrogenase B	40	VDR	Vitamin D Receptor

Supplementary Table S3. Candidate active ingredient of SYF extracts.

No.	Name	Structural formula
1	3-(3,4-Dihydroxyphenyl)-2-hydroxypropanoic acid	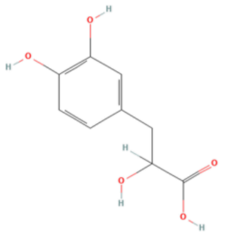
2	p-Coumaric acid	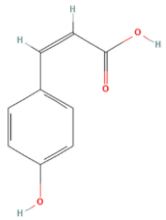
3	Kaempferol	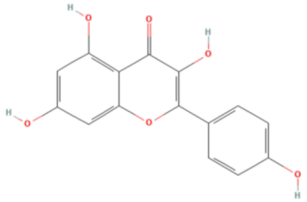
4	Vanillic_acid	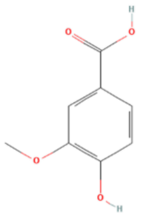

Supplementary Table S4. The main parameters and results of the molecular docking.

Compound names	Protein ID (PDB ID)	Hydrogen bonds (No)	Amino acid residues	Binding affinity (kcal/mol)
p-Coumaric acid	CA9 (1JCZ)	1	ARG240	-5.4
	KDM4C (2XDP)	2	HIS964, ARG919	-5.2
	MMP1 (1FBL)	3	ASN180, ARG214, VAL215	-6.5
	MMP2 (1CXW)	0		-4.4
	MMP9 (4WZV)	1	LEU188	-6.0
	STAT3 (6NJS)	1	THR515	-5.3
Kaempferol	AKR1C3 (4ZFC)	3	ASN167, GLN222, LYS270	-9.4
	CA9 (1JCZ)	1	ARG240	-7.8
	MMP2 (1CXW)	2	ARG34, TYR47	-5.9
	MMP9 (4WZV)	1	VAL223	-9.5
	MMP12 (1OS9)	1	TYR240	-9.8
	PTGS2 (1PXX)	1	TYR2130	-8.6
	STAT3 (6NJS)	2	ASP371, LEU438	-8.1
Vanilic acid	AKR1C3 (4ZFC)	1	TRY55	-6.3
	CA9 (1JCZ)	1	ILE242	-5.8
	KDM4C (2XDP)	1	TYR984	-5.2
	MMP1 (1FBL)	1	ALA182	-6.8
	MMP2 (1CXW)	1	ARG34	-4.2
	MMP9 (4WZV)	1	TRY245	-6.3
	STAT3 (6NJS)	2	ASN538, SER540	-5.1
3,4-Dihydroxyphenyllactic acid	STAT3 (6NJS)	0		-6.0

Supplementary Table S5. RT-qPCR primer information.

Genes	Forward primer sequence (5’-3’)	Reverse primer sequence (5’-3’)
Bcl-xL	GAGCTGGTGGTTGACTTTCTC	TCCATCTCCGATTCAGTCCCT
Cyclin D1	GCTGCGAAGTGGAAACCATC	CCTCCTTCTGCACA CATTTGAA
Jagged1	GTCCATGCAGAACGTGAACG	GCGGGACTGATACTCCTTGA
MMP2	CTCGGTAGGGACATGCTAAGTAGAG	CCTCTGGAGGTTCGACGTGA
MMP9	TCATGAGGAAGAGCTCTGAGT	TCATGAGGAAGAGCTCTGAGT
STAT3	CAGCAGCTTGACACACG	AAACACCAAAGTGGCATG
Survivin	CGACGTTGCCCCCTGCCTG	AAGGAAAGCGCAACCGGACGA
GAPDH	CGGATTTGGTCGTATTG	GAAGATGGTGATGGGATT
